# Characterizing Genetic, Epigenetic, Nutritional, and Clinico-Biochemical Profile of Women With Polycystic Ovary Syndrome: A Case–Control Study

**DOI:** 10.1155/jnme/8817919

**Published:** 2025-05-30

**Authors:** Woroud M.-Majd Chaker, Raghda Mohammed Adi, Mohamed Madkour, Nada Farhat, Dana N. Abdelrahim, Maha Saber-Ayad, Jibran Sualeh Muhammad, Ghada Mohammed, Noha Ahmed Mousa, Ameenah Saadi, Arathy Lakshmi, Mai Kazim, Nagla Elhadi Abdalla, Nariman Awad, Fatima AlAnouti, MoezALIslam E. Faris

**Affiliations:** ^1^College of Medicine, University of Sharjah, Sharjah, UAE; ^2^Department of Medical Laboratory Sciences, College of Health Sciences, University of Sharjah, Sharjah, UAE; ^3^Research Institute of Medical and Health Sciences, University of Sharjah, Sharjah, UAE; ^4^Clinical Sciences Department, College of Medicine, University of Sharjah, Sharjah, UAE; ^5^Faculty of Medicine, Cairo University, Cairo, Egypt; ^6^Department of Biomedical Sciences, College of Medicine and Health, University of Birmingham, Birmingham, UK; ^7^University Hospital of Sharjah, Sharjah, UAE; ^8^College of Natural and Health Sciences, Zayed University, Dubai, UAE; ^9^Department of Clinical Nutrition and Dietetics, Faculty of Allied Medical Sciences, Applied Science Private University, Amman, Jordan

**Keywords:** epigenetic modification, gene expression, precision medicine, precision nutrition, preproduction

## Abstract

**Objectives:** Polycystic ovary syndrome (PCOS) is among the most frequently encountered endocrinopathies in women. This study aimed to characterize the genetic (*INSR, FTO, DENND1A*, and *Nrf2*) expressions and epigenetic (DNA methylation) modifications, nutritional, metabolic, inflammatory, and hormonal markers for patients with PCOS in comparison with their age-matched healthy controls.

**Design & Methods:** The study also aimed to assess the genetic expressions concerning vitamin D status. Sixty-six patients with PCOS and 69 age-matched healthy controls were recruited. Fasting blood samples were used to measure genetic and biochemical variables. Real-time PCR was used to assess gene expressions, the bisulfite conversion method was used to evaluate DNA methylation, and multiplex immunoassays were used to measure inflammatory markers.

**Results:** Only two genes (*INSR and FTO*) were significantly (*p* < 0.05) upregulated, while one gene (*Nrf2*) was significantly (*p* < 0.05) downregulated in cases in comparison with controls. Furthermore, cases showed significantly (*p* < 0.05) higher BMI (kg/m^2^), fat mass, visceral fat surface area, and body fat percentage, as well as higher serum triglyceride levels, atherogenic index, VLDL levels, and TC/HDL and TG/HDL ratios when compared to controls. In contrast, HDL levels were significantly lower (*p* < 0.05) in the cases. Inflammatory markers (*hs-*CRP, IL-1β, IL-6, TNF-α, and VEGF) were significantly (*p* < 0.05) higher, while anti-inflammatory markers (IL-2 and IL-10) were significantly lower in cases when compared to controls.

**Conclusions:** Women with PCOS may have distinct genetic expressions and anthropometric, metabolic, and inflammatory markers that predispose to the progression of the disease. Identifying predictive biomarkers fosters the application of precision medicine and personalized nutrition approaches in preventing and managing PCOS.

## 1. Introduction

Polycystic ovary syndrome (PCOS) is a common hormonal and metabolic disorder among women of reproductive age, with an incidence ranging from 4% to 20%. The exact etiology of PCOS remains unknown despite long-standing research and numerous hypotheses [1]. There is evidence of its association with various ovarian cellular dysfunctions, disordered androgen synthesis or metabolism, disturbed gonadotrophin ratio, and insulin resistance (IR). PCOS frequently presents with variable menstrual or ovulation irregularities and clinical or biochemical hyperandrogenism. Central or abdominal obesity is one of the typical clinical features of PCOS, which in turn exacerbates IR. Women with PCOS are more likely to develop metabolic syndrome later in their lives, as well as impaired glucose tolerance, Type 2 diabetes (T2D), dyslipidemia, cardiovascular illnesses, and hypertension, in addition to the clinical and hormonal features [2, 3].

The severity of hyperinsulinemia and IR sufficiently impacts the phenotype of PCOS and is considerably regulated by the interplay between genetic (such as polymorphism in the insulin gene regulatory region) and environmental factors, particularly diet-related obesity [4]. Since environmental and hormonal changes directly affect clinical symptoms and PCOS development, epigenetic changes may also be crucial for PCOS outcomes and the clear hereditary foundation. Therefore, the development of PCOS may be influenced by epigenetic changes in the variety of clinical symptoms, environmental epigenetic disruptors, intrauterine hormonal and metabolic changes impacting embryo development, and adult lifestyle, which are considered significant in studying PCOS and the effect of various interventions [5].

Several genetic factors have been involved in the pathogenesis of PCOS. Among these, the fat mass obesity gene (*FTO*), which affects adiposity and the development of T2D, has emerged as a predisposing factor. *DENND1A* is another gene that is involved in the pathogenesis of PCOS. It was reported that an alternatively spliced form of *DENND1A* (*DENND1A.V2*) is remarkably increased in theca cells, the source of the excess androgens that characterize PCOS [6, 7]. The transcription factor (nuclear factor erythroid 2–related factor 2) (*Nrf2*) gene controls the formation of antioxidant proteins, protecting against oxidative stress (OS). Several genetic studies have related the *Nrf2* gene polymorphism (rs6721961) to OS-related illnesses such as respiratory disease, cardiovascular conditions, male infertility, and PCOS [8].

Imbalanced levels of anti-inflammatory and proinflammatory cytokines have been linked to ovarian dysfunction, steroidogenesis, and follicular maturation [9]. Numerous inflammatory indicators have recently been reported to be elevated in women with PCOS. White blood cell (WBC) count, high-sensitivity C-reactive protein (*hs-*CRP), and several cytokine concentrations, such as interleukin-6 (IL-6), IL-17, and tumor necrosis factor-α (TNF-α), are all elevated in these patients [10]. Moreover, chronic low-grade systemic inflammation has been associated with IR and abdominal obesity, as well as endothelial dysfunction, atherosclerosis, and coronary heart disease [11]. Additionally, a few researchers have reported the association of PCOS with low serum 25(OH)D or vitamin D deficiency [12]. Although several studies documented a link between PCOS and low-grade systemic inflammation, the attribution of the exact cause of PCOS to obesity and IR remains uncertain. It has already been suggested that hyperandrogenemia has proinflammatory effects and that the inflammatory state in women with PCOS is worse than that caused by IR and obesity alone [10]. Intriguingly, low vitamin D status adversely affects glucose homeostasis and is associated with obesity and IR [13, 14] and increased inflammatory status [15].

There is a knowledge gap pertaining to PCOS etiology among the UAE population because most of the available studies are only related to the prevalence of PCOS. A few research investigations have demonstrated PCOS's association with other diseases prevalent among the UAE population. Still, no case–control population-based studies have been conducted to examine the gene expression level and DNA methylation in conjunction with glycemic control and inflammatory, hormonal, and nutritional characteristics. These factors vary highly between different ethnicities, dietary, and lifestyle characteristics [16]. This study aimed to assess and characterize gene expressions, DNA methylation, metabolic, inflammatory, hormonal, and nutritional characteristics among patients with PCOS compared to the pattern among healthy women. More specifically, the current work aimed to examine the differences in the gene expressions, namely, the genes (*FTO, INSR, DENND1A,* and *Nrf2*) in the context of vitamin D status and epigenetic modifications (specifically in gene *INSR*) among a cohort sample of women within the UAE diagnosed with PCOS in comparison with the counterpart healthy controls.

## 2. Methods and Materials

### 2.1. Study Design and Setting

This research is an observational case–control study using quantitative data. It utilized the quantitative observational case–control study design, which examined the genetic expression profile and DNA methylation for specific genes. The study participants were recruited from the University Hospital of Sharjah, Obstetrics and Gynecology (OBG) Department, UAE, in collaboration with the OBG clinicians and with the assistance of the staff nurses.

### 2.2. Sample Size Calculation and Sampling Method

The sample size is calculated based on the assumption that PCOS's prevalence is approximately 5% and that the odds ratio (OR) is at least 2.0, with 80% power and a significance level of 0.05. In the current work, at least 60 women diagnosed with PCOS, alongside at least 60 age-matched healthy control women, were required for comparison between cases and controls. Subjects were interviewed to provide sufficient information about the study. Prior to participation, consent forms were signed by subjects who completed a general self-administered personal questionnaire and were exposed to anthropometric measurements.

### 2.3. Ethical Approval

The protocol of this study was designed and implemented in line with the guidelines specified by the Declaration of Helsinki. The survey's protocol and data collection instruments were reviewed and approved by the Research Ethics Committee at the University of Sharjah Hospital (UHS-HERC-118-08112022). The participants were given the right to withdraw from the study at any time and may refuse to provide/share any information they do not wish to. The data and samples collected will be only used for this research. Participants' identities were kept private and anonymous. If the participants had any queries, they were given contact information from the principal investigators and the ethical committee.

### 2.4. Diagnosis of PCOS, Inclusion, and Exclusion Criteria

Women aged 18 years or older who were diagnosed with PCOS met the inclusion criteria of this study. The diagnosis of PCOS was based on the Rotterdam criteria [17]; women with two of the following three criteria were considered to have PCOS: oligo-ovulation and anovulation, biochemical signs of hyperandrogenism, and polycystic ovaries on ultrasound examination (defined as the presence of 12 follicles measuring 2–9 mm in diameter and an ovarian volume 10 cm^3^).

Furthermore, participants had a stable diet and a high physical activity (PA) level throughout the study. This was stressed during the interview session and assured during the follow-up over the study period using personal communication and via WhatsApp group for participants.

#### 2.4.1. Exclusion Criteria

During the recruitment for the study, we excluded patients who were diagnosed with an androgen-secreting tumor, Cushing syndrome, congenital adrenal hyperplasia, hyperprolactinemia, pregnant or lactating women, women with diabetes, hypothyroidism, liver disease, renal dysfunction, or cardiovascular disease. Study participants who took medications known to affect metabolic parameters, such as antidiabetic drugs and calcium supplementation, were also excluded.

### 2.5. Data Collection

Questionnaires that included their sociodemographic information (age, nationality, marital status, education, occupation, and PA level) were filled out anonymously. Participants were given a brief description of their PA levels to facilitate describing their PA level. Anthropometric measurements (body weight and height) were taken using standard methods and assessment tools. Body composition compartments (body fat percentage, muscle mass, and fat-free mass) and visceral fat area were measured by bioelectrical impedance analysis (BIA) using a TANITA body composition analyzer. A registered phlebotomist has collected the blood sample. For IR, HOMA-IR was calculated using the following: as fasting plasma insulin (μU/mL) × fasting plasma glucose (FPG) (mg/dL)/405. As a measure of insulin sensitivity, QUICKI was calculated as 1/log fasting insulin (μU/mL) + log fasting glucose (mg/dL) [18].

### 2.6. Blood Sampling and Preparation

Blood was collected from each participant after fasting for at least 6–8 h. Laboratory analysis was conducted in a private laboratory in Sharjah, UAE. In the laboratory, serum 25-hydroxyvitamin D (25(OH)D), serum *hs*-CRP, and serum insulin (IDS, Boldon, UK) levels were determined using an ELISA kit, as per the manufacturer's instructions, and LDN, Nordhorn, Germany. An enzymatic kit was used to measure FPG. FSH, LH, and TSH were determined by using commercial kits (DiaMetra, Milano, Italy). A fully automated clinical chemistry analyzer (Adaltis, Pchem1, Italy) was used to quantify fasting glucose (FBG), total cholesterol (TC), low-density lipoprotein (LDL) cholesterol, high-density lipoprotein (HDL) cholesterol, and triglycerides (TG).

### 2.7. RNA Extraction, cDNA Synthesis, and DNA Extraction

QIAmp RNA Blood Mini kit (Qiagen, Cat. No. 52304) was used for RNA extraction per the manufacturer's instructions. RNA was then quantified using Nanodrop, and the Abs 260/280 ratio was observed to check the purity of the extract. RNA was stored at −80°C. To synthesize complementary DNA for qRT-PCR use, GoScript Reverse Transcription System (Promega. Cat. No. A5001) as per manufacturer's instructions. QIAmp DNA Blood Mini kit (Qiagen, Cat. No. 51104) was used for DNA extraction. First, 200 μL of packed cells in EDTA tubes was added to 20 μL of QIAGEN protease and 200 μL of buffer AL. Tubes were vortexed and then incubated at 56°C for 10 min. The remaining steps were followed as per the kit manufacturer's instructions. After DNA elution, it was quantified using Nanodrop, and the Abs 260/280 ratio was observed to check the purity of the extract. DNA was stored at −20°C [19].

### 2.8. Bisulfite Conversion and Quantitative Methylation-Specific PCR (qMSP)

For the qMSP analysis, the DNA samples were subjected to bisulfite conversion using the MethylEdge Bisulfite Conversion (Promega, Cat. No. N1301). A volume of 20 μL of gDNA was used in accordance with the manufacturer's instructions. The bisulfite-converted DNA was then utilized for methylation-specific PCR. We obtained two forward and reverse primers, methylated and unmethylated, for the *INSR* gene (Tables 1 and 2). The methylation percentage was calculated using the following formula: 1 − (Ct value of methylation/Ct value of methylation − Ct value of unmethylation). The results were then normalized by dividing them by the control percentage of methylation [20, 21].

### 2.9. Quantitative Real-Time PCR

GoTaq qPCR Master Mix (Promega, Cat No. A6001) was used to perform real-time PCR. All reactions were performed on ice in a biosafety cabinet. Forward and reverse primers (Table 1) were diluted 1:10 with nuclease-free water. PCR tubes were labeled and inserted on the rack, and the following reaction components were added: 7 μL of nuclease-free water, 1 μL of diluted forward primer, 1 μL of diluted reverse primer, 1 μL of sample cDNA, and 10 μL of GoTaq qPCR Master Mix, 2x, which made the total volume 20 μL. All samples were done in triplicates. No template control that contained the same components except cDNA was added. Tubes were mixed gently and spun down to be incubated in a QuantStudio 3 machine for 40 cycles. Ct values were used to calculate Expression Fold Change 2^−ΔCt^ [22].

### 2.10. Multiplex Immunoassay

The multiplex technique was used to analyze plasma proinflammatory and anti-inflammatory cytokines. The Human Cytokine A Premixed Magnetic Luminex Performance Assay (R and D System, Inc., Minneapolis, USA) was used, following the instructions. The assay detected various cytokines, including IL-1α, IL-2, IL-6, IL-8, IL-10, IL-17, TNF-α, and vascular endothelial growth factor (VEGF).

### 2.11. Statistical Analysis

This study employed a case–control design. The continuous variables were described as mean ± standard deviation (SD), and then, cases and controls were compared using an independent sample t-test. Body mass index (BMI) was calculated based on weight and height and then described by WHO into four categories: underweight, average weight, overweight, and obese. All of the sociodemographic data were expressed as frequencies and percentages. New variables were initiated based on valid calculations, and then, a comparison of cases and controls was conducted. The 25(OH)D level was measured in ng/mL and then converted to nmol/L.

T-tests were used to compare cases and control groups based on their results in glucoregulatory markers, lipid profile markers, 25(OH)D, hormonal markers, inflammatory markers, and gene expression markers, and data are represented as mean ± SD. The cutoff points were applied to the glucoregulatory markers, lipid profile markers, serum 25(OH)D, and hormonal markers. Then, the categorical variables were described as frequencies and percentages of observed values, and the comparison between case and control was done using Crosstab and chi-square test.

Logistic regression was performed, OR was calculated, and significance was considered at *p* value < 0.05. All data were encoded, and all analyses were performed using IBM SPSS statistics, version 27.0 (USA). All the data significance level was set at a *p*  < 0.05 for all data. The graph's statistical analysis was performed using GraphPad Prism (version 8.4.2) software.

## 3. Results

### 3.1. Patient Characteristics

Sixty-six patients with PCOS and 69 healthy controls were enrolled in this study. The sociodemographic data of the study are shown in Table 3. There was no significant age difference between cases and controls, indicating proper matching, and no significant difference in the educational level between cases and controls. The vast majority of the cases were Arabs. The cases were higher in the nonmedical occupation, whereas the controls were higher in the medical field. The incidence of PCOS was lower among the medical professions than among nonmedical professions. The high and moderate incomes were more reported among the cases, while the lower income was more reported among the controls, with significant variations. PCOS was more common among married women than single women (Table 3).

### 3.2. Health and Medical Data

The cases exhibited significantly irregular menstrual periods (50.0%) compared to the control group (13.0%) (*p*=0.001). In the cases, OBG surgical history operation was higher (37.9%) than controls (23.2%, *p*=0.047). Compared to controls, the cases presented significantly higher intake of dietary supplements (herbal extracts, botanicals, omega-3, multivitamins, and minerals) (14.5% and 27.3%, respectively, *p*=0.052) and herbal teas and boiled seeds (saffron, curcumin, flaxseed, turmeric, nettle, milk thistle) (11.6% and 24.2%, respectively, *p*=0.044). There was no significant difference in sun exposure between cases and controls. There were no significant differences in the use of vitamin D supplements nor the serum levels of 25(OH)D between the cases and controls. Implies that other dietary, lifestyle factors or genetic predisposing factors are associated with the development and progression of the disease. No differences in the daily vitamin D intake and number of pregnancies were reported between the cases and controls (Table 4).

### 3.3. Body Anthropometrics

The case group had a significantly greater mean weight, BMI, mean muscle mass, mean visceral fat surface, mean body fat percentage, and mean FFMI than the controls (*p*=0.001) (Table 5). The cases also had a significantly higher mean fat mass (*p*=0.002) (Table 5). The BMI (kg/m^2^) distribution shows that the case group had significantly higher percentages of individuals classified as overweight and obese (71.2%) than the control group (28.9%) (*p*=0.001) (Table 5).

### 3.4. Glucoregulatory Markers

Glycemic control markers of the study sample revealed the lack of statistically significant variations in serum glucose, insulin, and HOMA-IR between patients and controls. Even so, there were apparent trends for higher insulin, IR, and serum glucose levels in cases compared to healthy controls. Nonetheless, there were significantly higher insulin sensitivity QUICKI scores among healthy controls than cases (*p*=0.038) (Table 6).

### 3.5. Lipid Profile and Atherogenic Markers

The HDL levels were significantly lower in the cases compared to the healthy controls (*p*=0.001). On the other hand, TG (*p*=0.005), atherogenic index (*p*=0.001), VLDL (*p*=0.005), and TG/HDL (*p*=0.001) levels were significantly higher in the cases compared with healthy controls (Table 6).

### 3.6. Vitamin D and Hormonal Profile

No significant differences between cases and controls regarding vitamin D, FSH, TSH, LH, and LH/FSH ratio were found. Still, there were clear trends toward having lower TSH, LH, and LH/FSH ratios in the cases compared to the healthy controls (Table 6).

### 3.7. Inflammatory Markers

As shown in Table 6, cases showed significantly higher levels of the inflammatory marker (*hs-*CRP, *p*=0.002) and the proinflammatory markers IL-1*α*, IL-6, IL-17 (*p*=0.001), TNF-α (*p*=0.005), and VEGF (*p*=0.025) than controls. The healthy controls showed significantly higher levels of the anti-inflammatory cytokines (IL-2 and IL-10, *p*=0.001).

### 3.8. Categorical Biochemical Variables

Supporting Information Table 1 shows the categorical laboratory results for the glucoregulatory markers, lipid profile, 25(OH)D, and hormonal markers. In the glucoregulatory markers, albeit not significantly different, cases showed numerically higher values for serum glucose, serum insulin, and IR (HOMA-IR) values than healthy controls. Regarding the lipid profile, notwithstanding not being statistically different, cases showed a generally higher prevalence of high TC, TG, LDL, and lower HDL levels than their counterparty healthy controls. Furthermore, the majority of the study participants, both cases and controls, were vitamin D deficient (59.1% and 56.5%, respectively), with 25(OH)D levels below 20 ng/mL (below 50 nmol/L) abundantly found both in cases and controls, without being statistically different. Albeit not significantly different, a trend was observed toward lower 25(OH)D levels among cases, with deficient and insufficient vitamin D status being more prevalent in PCOS patients (83.3%) than controls (72.4%).

### 3.9. Gene Expressions

**Figure 1:**
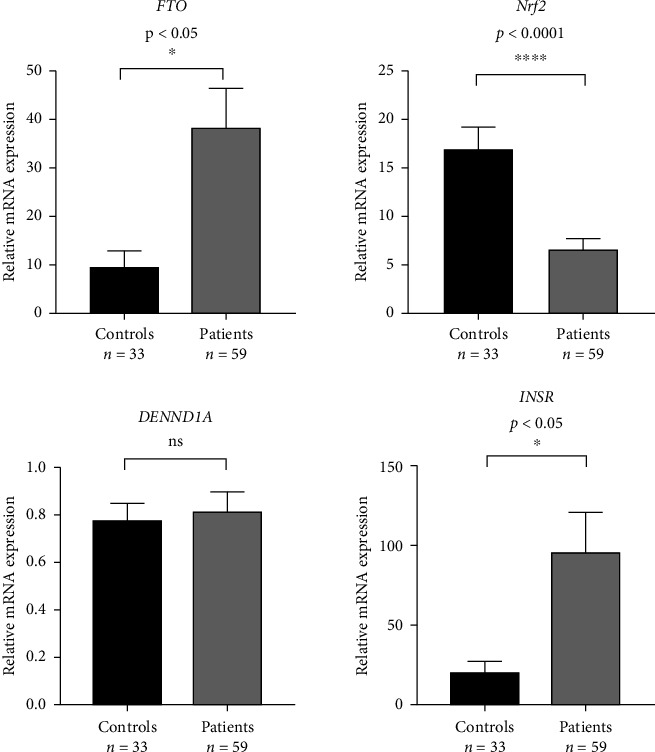
Levels of expression for the genes in PCOS cases and control groups.

**Figure 2:**
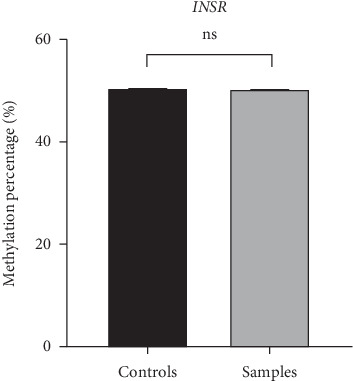
Methylation percentage (%) for the *INSR* gene in PCOS cases and control groups.

As depicted in Figure 1, the *FTO* gene was overexpressed when compared with the level of gene expression among the healthy controls (*p*  <  0.001). It was about four times greater among the patients with PCOS. For the *INSR* gene, the relative expression of the *INSR* gene among patients with PCOSs and controls reveals a highly significant difference (*p*  < 0.05). In the PCOS group, the expression of the *INSR* gene was overexpressed when compared with the gene expression level among the healthy controls. It was about four times greater among the patients with PCOS in comparison with the healthy counterpart controls. Although *INSR* gene expression is significantly higher in many patient samples, the MSP data show no significant difference in methylation levels in control versus patient samples (Figure 2). The DENND1A gene expression was not significantly upregulated in patients with PCOS compared to the control group (Figure 1).

Regarding gene expression fold change of the gene *Nrf2,* there was a highly statistically significant difference between the expression of the gene *Nrf2* among patients with PCOS compared to the healthy controls (*p*  < 0.0001). In the PCOS group, the expression of the *Nrf2* gene was drastically downregulated compared with the counterpart healthy group, implying that patients with PCOS have excessively higher rates of OS and inflammation (Figure 1).

### 3.10. Regression Analysis

Table 7 shows the multiple linear regression analysis for serum 25(OH)D for vitamin D status categories with the gene expression markers in both patients and control individuals. Several studies were performed to investigate the relationship between the predictive variables (gene expressions) and the outcome-dependent variables (serum 25(OH)D levels for vitamin D status categories; deficient, insufficient, and sufficient). The *Nrf2* significantly predicts the insufficient serum level of 25(OH)D, with those diagnosed with inadequate vitamin D status having 40% lower expression for the *Nrf2* gene expression (*p*=0.018).

## 4. Discussion

The current study aimed to elaborate on the different characteristics that differentiate a cohort of patients with PCOS from their counterpart healthy controls from the UAE population recruited in Sharjah. These characteristics include the genetic and epigenetic (DNA methylation), metabolic, inflammatory, nutritional, and hormonal markers between the cases and controls in a group of women in Sharjah/UAE.

Our study revealed that significant upregulations (*FTO* and *INSR*) and downregulation (*Nrf2*) in specific genes were reported among PCOS patients compared to their healthy counterpart controls. Significant differences were noted between the cases and controls regarding the body anthropometrics, part of glycemic control markers, lipid profile and atherogenic factors, and the inflammatory/proinflammatory markers. The majority of individuals, both patients and control subjects, were vitamin D deficient, with vitamin D levels below 20 ng/dL in 59.1% of cases and 56.5% of controls and a mean serum level of 56.10 ± 48.17 nmol/L for cases and 57.47 ± 38.67 nmol/L for healthy controls. The high prevalence of vitamin D deficiency among both groups, together with previous studies [23, 24], calls for a large population study to define the normal 25(OH)D levels in the MENA region countries, including UAE.

Most of the participants in the study were Arabs, as the study was conducted in the Arabic region of Sharjah and one hospital. Cases of PCOS were more common among nonmedical professionals than medical, which could be related to their better background knowledge of the disease risk factors and more ease of access to medical information pertaining to the disease etiology and prevention. Our data also suggest that PCOS may be more prevalent in high-income individuals, as they may have better access to healthcare services and may, therefore, be more likely to be diagnosed with PCOS [25]. Additionally, unhealthy lifestyle behaviors that are more common among high-income individuals, such as sedentary lifestyles and smoking, have been associated with an increased risk of PCOS [26, 27]. This relationship could also be explained by the fact that higher income in the UAE may be associated with more frequent intake of unhealthy energy-dense and low nutrient-dense fast foods from restaurants and delivery services [28, 29]. Such a matter may act as a predisposing factor for the pathogenesis of obesity and the subsequent metabolic derangements accompanying PCOS. Zhang et al. found that the prevalence of central obesity (as measured by waist:hip ratio [WHR]) was doubled in Chinese patients with PCOS compared to healthy controls, with PCOS patients having a higher WHR and a larger trunk/periphery fat ratio, all of which indicate central obesity [30]. Also, PCOS appears to be more prevalent in married women, most likely because of more frequent diagnoses among this group, who are more prone to go for medical check-ups, especially with the lack of fertility among those women in the UAE society [31].

The reported lack of differences in the use of vitamin D supplements, the serum levels of 25(OH)D, and the amount of daily vitamin D intake between the cases and controls imply that other dietary, lifestyle factors or genetic predisposing factors are associated with the development and progression of the disease [32, 33].

The current research examined gene expression for four candidate genes (*FTO, INSR, DENND1A*, and *Nrf2*) and DNA methylation for one gene (*INSR*) involved in the pathogenesis of PCOS among women with PCOS in the UAE in relation to vitamin D status. Two genes (*FTO and INSR*) were profoundly upregulated. At the same time, one was downregulated (*Nrf2*) among the patients with PCOS compared to their counterpart healthy controls, with the gene *Nrf2* only showing significantly lower expression among vitamin D insufficient women with PCOS.

Fat mass and obesity-associated (*FTO*) genes are primarily expressed in hypothalamic nuclei, which play a crucial role in energy balance, body mass regulation, and nucleic acid demethylation. Overexpression of *FTO* has a significant impact on the risk of PCOS, primarily by its role in increasing BMI and secondarily through subsequent metabolic derangement, including IR and hyperandrogenemia [34]. Dysregulation of the *FTO* gene has been linked to various conditions, including obesity, T2D, and PCOS, which contributes to these conditions through its effect on body weight and BMI, in addition to its impact on metabolic factors like IR, insulin sensitivity, serum glucose, and hyperandrogenemia [34–36]. The current study reported a significant correlation between *FTO* expression among patients with PCOS and BMI in body fatness (mass and percent) and visceral fat levels. In addition, upregulated *FTO* expression was also associated with higher serum glucose, insulin levels, and IR among the patients with PCOS compared with the healthy control and significantly lower QUICKI insulin sensitivity among the cases compared with the healthy controls. This also concords with the existing literature, showing that patients with PCOS have higher BMI [37], higher serum glucose, insulin, IR, and lower insulin sensitivity [34]. Furthermore, three case–control studies conducted among women of various races with PCOS revealed the association of the *FTO* gene and PCOS disease, demonstrating that the *FTO* gene was mainly linked to high BMI and elevated anthropometric parameters and indicating that different ethnicities with different dietary habits and lifestyle behaviors may contribute and to the progression of PCOS [38–40].

The FTO protein plays a role in the development of obesity by influencing the N6-methyladenosine (m6A) level in hormones involved in eating or adipogenesis. This protein is responsible for demethylating RNA molecules through a hydroxylation reaction that requires molecular oxygen and α-ketoglutarate as cofactors. The hydroxylation of RNA molecules by FTO protein affects the stability and efficiency of translation of the targeted mRNA [41]. Dysregulation of the *FTO* gene has been linked to various conditions, including obesity, T2D, and PCOS, through its direct effect on body weight and BMI, in addition to its impact on metabolic factors like IR, insulin sensitivity, serum glucose, and hyperandrogenemia [34–36].

The insulin receptor gene *(INSR)* encodes a member of the receptor tyrosine kinase family of proteins. Firstly, a preproprotein is formed, then proteolytically processed to generate alpha and beta subunits that produce a hetero-tetrameric receptor. The binding of insulin or other ligands (including insulin-like growth factor) to this receptor activates the insulin signaling pathway, thus regulating glucose uptake and release and several metabolic cellular processes. Mutations of *INSR* lead to the inherited severe IR syndromes [42]. In our study, there was no significant difference in methylation levels in patient versus control samples, suggesting that although the expression of the *INSR* gene might be related to PCOS pathology, the *INSR* gene is not regulated by DNA methylation in these patients. Hence, despite being prone to promoter CpG methylation, the *INSR* gene in PCOS patients is not regulated by DNA methylation-based epigenetic changes, and other mechanisms might be involved.

PCOS is a chronic systemic condition associated with chronic inflammation and OS. CRP is a general inflammatory parameter and one of the most sensitive predictors of cardiovascular diseases [43]. In our study, the data revealed significantly higher levels of CRP in women with PCOS when compared to their healthy counterpart controls. This was consistent with Blumenfeld, who reported significantly higher CRP levels in PCOS patients versus controls [44]. In addition, Makedos et al. demonstrated that serum levels of *hs*-CRP concentrations were more significant in normal-weight women with PCOS than in normal-weight women without PCOS [43]. PCOS is typically accompanied by hormonal irregularities depicted by increases in luteinizing hormone (LH), prolactin, estrogen, and serum androgens (testosterone and androstenedione). Hormonal measurements show that many women with PCOS have an elevated LH/FSH ratio [45]. The present study showed a clear trend toward lower TSH, LH, and LH/FSH ratios in cases compared with the control group. However, Christodoulopoulou et al. found that those women with menstrual problems had significantly greater levels of LH and TSH than those with regular menstrual cycles [46].

The present study showed no significant differences between the cases and controls in the glycemic markers (serum glucose, insulin, HOMA-IR, and QUICKI insulin sensitivity). In concordance, Christodoulopoulou et al. demonstrated that none of the glucose control indicators (QUICKI, HOMA-IR) were substantially different in the group with menstrual problems [46]. Although there were no statistically significant variations in glucose, insulin, or the HOMA-IR score between patients and controls, there is a noticeable trend in detecting a higher prevalence of IR in cases versus control (33.3% moderate-to-severe resistance in cases and 21.7% in controls). Our overall results are supported by the findings of the study of Talbott et al. (1998), where the women with PCOS reported a substantial rise in BMI, LDL, atherogenic index, WHR, fasting insulin, systolic blood pressure, and reduced levels of total HDL when compared to controls [47].

Our findings indicate that PCOS patients had a higher prevalence of irregular menstrual periods, previous surgical operations, especially cesarian surgeries, consumption of herbal teas and boiled seeds (saffron, curcumin, flaxseed, turmeric, nettle, milk thistle), supplements (herbal extracts, botanicals, omega-3, multivitamins, and minerals), and use of PCOS medication, and other study results were consistent with our findings. A previous study found that women with PCOS were more likely to have irregular menstrual cycles compared to women without PCOS [45]. Another study found that women with PCOS had a higher prevalence of previous surgical operations, including lower segment cesarean section (LSCS), compared to women who had no PCOS [13]. In addition, another study found that women with PCOS were more likely to consume edible seeds and herbs compared to women without PCOS [48]. This is most likely because PCOS patients are increasingly turning to herbal therapy as an alternative to synthetic drugs for the control and treatment of PCOS and to improve recovery rates and pain management with better acceptability [48], and it may be less costly.

*Nrfs2* is a vital transcription factor that induces the expression of many cytoprotective and antioxidant proteins, thus protecting against OS and resulting damage. The *Nrf2*–antioxidant response element (ARE) pathway is an intrinsic defense mechanism against OS [49]. The reported downregulation of the *Nrf2* gene among patients with PCOS indicates an increased rate of OS and inflammation, which contribute to the development of the disease. In our study, this was concordant since the CRP levels were significantly higher among the patients with PCOS compared to their healthy counterpart controls, which is consistent with previous studies [50–52].

Intriguingly, *Nrf2* gene polymorphism (rs6721956) was significantly associated with PCOS, as revealed by a study conducted on Pakistani patients. Such genotype of *Nrf2* is characterized by the downregulation of the expression of antioxidant enzymes that suppress the OS, which predisposes to PCOS [53]. The *Nrf2* gene regulates the expression of several antioxidant and detoxification genes by binding to the ARE in their promoter region. Under normal conditions, *Nrf2* is sequestered in the cytoplasm by the Kelch-like ECH-associating protein 1 (Keap1), leading to proteasomal degradation. In situations with high OS, *Nrf2* separates from Keap1 and translocates to the nucleus, which links with ARE and triggers the activation of genes responsible for antioxidant and detoxification functions. This ultimately results in protecting the cells from any harm caused by OS. Thus, the *Nrf2* pathway has been demonstrated to be dysregulated in several pathologies, such as PCOS, resulting in elevated levels of OS and inflammation [54].

In the same context, a case–control study on 85 women diagnosed with PCOS demonstrated significantly low levels of serum antioxidant levels of glutathione, vitamins C and E, and notably increased activities of antioxidant enzymes such as glutathione peroxidase, glutathione reductase, and glutathione transferase, in comparison with those without PCOS [51]. Concurrently, a case–control study conducted among 51 Omani women diagnosed with PCOS showed lower levels of the antioxidant compound glutathione and total antioxidant capacity in PCOS women compared to the control group, with a statistically significant difference observed for GSH levels [52]. This finding supports our findings and aligns with the implication that OS could be involved in the progression of PCOS, suggesting that measuring OS parameters could be useful as diagnostic markers for identifying individuals at high risk of developing the condition.

The patients with PCOS exhibit an apparent increase in OS and inflammation and a decrease in the expression of the critical antioxidant gene *Nrf2*. Additionally, there is an increase in the expression of genes related to OS and inflammation. Therefore, it is necessary to intervene with dietary and lifestyle modifications, such as physical exercise and intermittent fasting. These modifications have been demonstrated to improve the proinflammatory cytokines [55], mitigate the OS condition [56, 57], improve body composition [58] and glucometabolic regulation [59], lower visceral adiposity [60], and balance the gene expressions implicated in the pathogenesis of PCOS such as *Nrf2* and *FTO* [61, 62].

Furthermore, previous research studies have consistently shown a direct correlation between low levels of vitamin D and reduced expression of the *Nrf2* gene [63]. This relationship aligns with our current finding, as well as the findings of multiple studies, both in humans and animals, which have demonstrated that optimal levels of 25(OH)D enhance the expression of *Nrf2* [64, 65].

### 4.1. Strengths and Limitations

There are several strengths in this study. This is the first case–control study conducted on PCOS patients in the UAE, reflecting distinct dietary and lifestyle behaviors within unique cultural and socioeconomic contexts. Furthermore, our research mainly depended on objective parameters, which minimizes the recall bias that characterizes the observational studies, including the case–control study. The comprehensive assessment of the environmental exposure factors such as vitamin D status, dietary supplement intakes, herbal tea use, sun exposure, and smoking that may impact the occurrence/severity of PCOS make the study more elaborate and informative. Also, the study's design is less costly and less time-consuming than other designs. Finally, the study paves the way to test hypotheses through future research.

The current study has a few limitations to consider when interpreting its results. First, the case–control design has inherent limitations, and the absence of intervention in this observational study makes it challenging to infer concrete associations or causality. Data were collected from a relatively small cohort at one hospital in Sharjah, using a convenient sampling technique, which limits the generalizability of the results; there may be a selection bias; and the studied cohort may not be representative of all ethnic groups in the UAE. Another limitation is that a small number of variables were dependent on recall, which poses a risk of recall bias. Lastly, PA was self-described by the study participants, which entails significant inaccuracy. More objective measurements for PA give a more accurate relationship between exercising and the progression of PCOS.

## 5. Conclusions

The pathogenesis of PCOS is associated with distinct anthropometric, inflammatory, metabolic, and genetic signatures. Future well-controlled, longitudinal intervention studies are warranted to find out the effect of different dietary and lifestyle interventions in the management and prevention of PCOS among women in the UAE. In our research on adult women with PCOS, the patients exhibited unique genetic expressions (upregulation of *FTO* and *INSR* and downregulation of *Nrf2*), along with specific metabolic, inflammatory, and anthropometric markers that make them more susceptible to the advancement of the condition. This underscores the need to incorporate PCOS into personalized medicine and personalized nutrition strategies when addressing the management of this disorder, with the ultimate goal of optimizing patient care.

## Figures and Tables

**Table 1:** Primers used in qRT-PCR and their base sequence.

Primer	Sequence
*DENND1A*—forward	TCCAGGATCAAGCAGAATCCA
*DENND1A*—reverse	TAGTCCTCCGGGAATTGCT
*FTO*—forward	GCTGCTTATTTCGGGACCTG
*FTO*—reverse	AGCCTGGATTACCAATGAGGA
*INSR*—forward	AAAACGAGGCCCGAAGATTTC
*INSR*—reverse	GAGCCATAGACCCGGAAG
*NRF2*—forward	TCCAGTCAGAAACCAGTGGAT
*NRF2*—reverse	GAATGTCTGCGCCAAAAGCTG

**Table 2:** Primers used in qMSP and their base sequence.

*INSR* MF	GATTCGGTTAGGGGACGTAT
*INSR* MR	ACGCCGATAACTTACGAACC
*INSR* UF	AGATTTGGTTAGGGGATGTAT
*INSR* UR	TTCCACACCAATAACTTACAAACC

**Table 3:** Description of sociodemographic and anthropometric data of the study sample comparison between cases and controls (*N* = 135).

Variable		Cases (*n* = 66) *n* (%)	Controls (*n* = 69) *n* (%)	*p* value (difference between cases and controls)
Age (years)	Mean ± SD	29.23 ± 7.25	28.62 ± 8.51	0.658

BMI (kg/m^2^)	Mean ± SD	28.83 ± 5.92	23.86 ± 5.15	**0.001** ^∗^

Nationality	Arabs	50 (75.8%)	34 (49.3%)	**0.001** ^∗^
	Non-Arab	16 (24.2%)	35 (50.7%)	

Education	Graduated high school	13 (19.7%)	12 (17.4%)	0.685
	Diploma	5 (7.6%)	3 (4.3%)	
	BSc	40 (60.6%)	48 (69.6%)	
	MSc	8 (12.1%)	6 (8.7%)	

Occupation	Medical	26 (39.4%)	49 (71.0%)	**0.001** ^∗^
	Nonmedical	40 (60.6%)	20 (29.0%)	

Income (AED/month)	< 10,000 (low)	52 (78.8%)	65 (94.2%)	**0.002** ^∗^
	10,000–20,000 (medium)	5 (7.6%)	4 (5.8%)	
	> 20,000 (high)	9 (13.6%)	—	

Smoking	Yes	3 (4.5%)	3 (4.3%)	0.762
	Occasionally	6 (9.1%)	4 (5.8%)	
	No	57 (86.4%)	62 (89.9%)	

Self-reported physical activity level	Very active	8 (12.1%)	12 (17.4%)	0.346
	Moderately active	53 (80.3%)	48 (69.6%)	
	Not active	5 (7.6%)	9 (13.0%)	

Marital status	Single	28 (42.4%)	46 (66.7%)	**0.005** ^∗^
	Married	34 (51.5%)	23 (33.3%)	
	Others (widowed, divorced)	4 (6.1%)	—	

*Note:* The comparison between cases and controls was made using an independent sample *t*-test in SPSS. Less than 0.05 values presented as bold and asterisked.

**Table 4:** Health and medication status of the study sample (*N* = 135).

		Cases (*n* = 66) *n* (%)	Controls (*n* = 69) *n* (%)	*p* value (difference between cases and controls)
*Categorical variables*				
Menstrual period	Regular	33 (50.0)	60 (87.0)	**0.001** ^∗^
	Irregular	33 (50.0)	9 (13.0)	
History of previous illness	Yes	11 (16.7)	9 (13.0)	0.632
	No	55 (83.3)	60 (87.0)	
History of OBG surgical operation	Yes	25 (37.9)	16 (23.2)	**0.047** ^∗^
	No	41 (62.1)	53 (76.8)	
Supplements (herbal extracts, botanicals, omega-3, multivitamins, and minerals)	Yes	18 (27.3)	10 (14.5)	**0.052** ^∗^
	No	48 (72.7)	59 (85.5)	
Herbal teas and boiled seeds (saffron, curcumin, flaxseed, turmeric, nettle, milk thistle)	Yes	16 (24.2)	8 (11.6)	**0.044** ^∗^
	No	50 (75.8)	61 (88.4)	
Exposure to the sun at least 10–30 min, 3–5 times a week	Yes	25 (37.9)	26 (37.7)	0.981
	No	41 (62.1)	43 (62.3)	
Vitamin D supplement	Yes	28 (42.4)	21 (30.4)	0.148
	No	38 (57.6)	48 (69.6)	

*Continuous variables*				
Dose of vitamin D (μg/day)		11,189.39 ± 18,902.54	8467.39 ± 18,301.63	0.393
Number of pregnancies		1.03 ± 1.46	0.80 ± 1.60	0.430

*Note:* The distribution of the case and control participants among different categories was assessed using the chi-square test in SPSS. Less than 0.05 values presented as bold and asterisked.

**Table 5:** Anthropometrics of the study sample (*N* = 135).

		Cases (*n* = 66) Mean ± SD	Controls (*n* = 69) Mean ± SD	*p* value (difference between cases and controls)
*Continuous variables*				
Weight (kg)		73.87 ± 16.06	64.79 ± 13.98	**0.0011** ^∗^
Height (m)		1.60 ± 0.06	1.65 ± 0.01	**0.001** ^∗^
BMI (kg/m^2^)		28.83 ± 5.92	23.86 ± 5.15	**0.001** ^∗^
Muscle mass (kg)		44.30 ± 6.15	40.66 ± 6.26	**0.001** ^∗^
Fat mass (kg)		27.57 ± 10.99	21.91 ± 9.21	**0.002** ^∗^
Visceral fat surface area (cm^2^)		57.46 ± 32.13	39.13 ± 25.42	**0.001** ^∗^
Body fat percent (%)		38.57 ± 7.99	31.50 ± 7.73	**0.001** ^∗^
Fat-free mass (kg)		44.22 ± 4.75	43.35 ± 3.82	0.246
Fat-free mass index (FFMI, kg)		17.24 ± 1.31	15.96 ± 1.39	**0.001** ^∗^

*Categorical variable*				
BMI (kg/m^2^)	Underweight (< 18.0)	1 (1.5%)	7 (10.1%)	**0.001** ^∗^
	Normal weight (18.5–24.9)	18 (27.3%)	42 (60.9%)	
	Overweight (25.0–29.9)	22 (33.3%)	13 (18.8%)	
	Obese (> 30)	25 (37.9%)	7 (10.1%)	

*Note:* The comparison between cases and controls was made using an independent sample *t*-test in SPSS. Less than 0.05 values presented as bold and asterisked.

Abbreviations: BMI = body mass index, FFMI = fat-free mass index.

**Table 6:** Glycemic control, lipid profile, serum 25(OH)D, hormonal and inflammatory markers of the study populations, and case–control groups (*N* = 135).

Variable	Cases (*n* = 66) Mean ± SD	Controls (*n* = 69) Mean ± SD	*p* value (difference between cases and controls)
Glucoregulatory markers			
Glucose (mg/dL)	87.35 ± 48.02	76.38 ± 16.85	0.078
Insulin (μIU/mL)	16.31 ± 16.53	12.06 ± 14.29	0.114
HOMA-IR (insulin resistance)	4.41 ± 7.49	2.57 ± 4.21	0.080
QUICKI (insulin sensitivity)	0.34 ± 0.05	0.36 ± 0.04	**0.038** ^∗^
Lipid profile and atherogenic markers			
LDL (mg/dL)	122.80 ± 31.28	146.36 ± 245.11	0.440
HDL (mg/dL)	53.84 ± 11.09	61.15 ± 11.44	**0.001** ^∗^
TG (mg/dL)	133.63 ± 79.15	100.46 ± 51.52	**0.005** ^∗^
TC (mg/dL)	202.52 ± 37.76	198.19 ± 36.33	0.501
VLDL (mg/dL)	26.73 ± 15.83	20.09 ± 10.30	**0.005** ^∗^
LDL/HDL ratio	2.36 ± 0.71	2.39 ± 3.74	0.941
TG/HDL ratio	2.72 ± 2.04	1.73 ± 1.04	**0.001** ^∗^
Atherogenic index	0.34 ± 0.28	0.17 ± 0.24	**0.001** ^∗^
25(OH)D and hormonal markers			
Serum 25(OH)D (nmol/L)	56.10 ± 48.17	57.47 ± 38.67	0.857
FSH (mIU/mL)	6.81 ± 8.25	6.91 ± 7.35	0.939
TSH (μIU/mL)	1.95 ± 1.32	2.17 ± 1.34	0.326
LH (mIU/mL)	8.03 ± 7.56	9.46 ± 13.35	0.451
LH/FSH ratio	1.35 ± 1.15	1.48 ± 1.12	0.533
Inflammatory markers			
*hs-*CRP (mg/dL)	4.65 ± 4.54	2.47 ± 3.20	**0.002** ^∗^
IL-1*α* (pg/mL)	11.82 ± 1.94	8.82 ± 1.21	**0.001** ^∗^
IL-2 (ng/mL)	17.30 ± 4.07	21.74 ± 3.58	**0.001** ^∗^
IL-6 (ng/mL)	2.33 ± 0.54	1.71 ± 0.52	**0.001** ^∗^
IL-8 (ng/mL)	47.32 ± 90.51	25.75 ± 30.61	0.104
IL-10 (ng/mL)	0.95 ± 0.17	1.30 ± 0.26	**0.001** ^∗^
IL-17 (ng/mL)	17.74 ± 2.83	13.91 ± 1.75	**0.001** ^∗^
TNF-α (ng/mL)	3.55 ± 1.64	2.75 ± 0.96	**0.005** ^∗^
VEGF (pg/mL)	80.44 ± 72.08	52.70 ± 42.22	**0.025** ^∗^

*Note:* The comparison between cases and controls was done using an independent sample *t*-test in SPSS. HOMA-IR index = homeostatic model assessment of insulin resistance, QUICKI = quantitative Insulin sensitivity check index. Less than 0.05 values presented as bold and asterisked.

Abbreviation: *hs-*CRP = high-sensitivity C-reactive protein.

**Table 7:** Multiple linear regression analysis between vitamin D status categories and gene expressions for patients with PCOS and healthy controls (*N* = 135).

Independent variables	Dependent variable (vitamin D status)								
	Deficient (*n* = 79)			Insufficient (*n* = 27)			Sufficient (*n* = 29)		
	OR	*p*	95% CI	OR	*p*	95% CI	OR	*p*	95% CI
Gene expression markers									
Cases									
*FTO*	0.092	0.304	−1.402, 0.835	0.311	0.258	−0.515, 1.760	0.375	0.256	−26.746, 8.088
*Nrf2*	0.114	0.263	−1.936, 3.707	0.599	**0.018** ^∗^	−4.832, −0.531	0.228	0.501	−27.468, 52.139
*DENND1A*	0.077	0.336	−2.356, 3.607	0.490	0.063	−7.043, 0.221	0.167	0.623	−66.470, 105.12
*INSR*	0.104	0.282	−1.112, 0.618	0.330	0.230	−0.470, 1.787	0.338	0.309	−23.051, 8.173
Controls									
*FTO*	0.258	0.151	−3.182, 1.050	0.556	0.195	−2.382, 8.997	0.272	0.514	−4.635, 8.298
*Nrf2*	0.193	0.254	−4.134, 7.893	0.177	0.704	−9.095, 6.636	0.769	0.074	−1.492, 20.978
*DENND1A*	0.024	0.462	−6.614, 6.038	0.487	0.268	−5.759, 16.613	0.379	0.402	−28.192, 13.382
*INSR*	0.361	0.070	−2.938, 0.456	0.402	0.372	−3.162, 7.065	0.431	0.286	−3.310, 9.389

*Note:* Multiple linear regression. Less than 0.05 values presented as bold and asterisked.

## Data Availability

The data that support the findings of this study are available upon request from the corresponding author. The data are not publicly available due to privacy or ethical restrictions.

## Supporting Information

Supporting Information Table 1: Subgroup differences between cases and controls.
