# Supplementary material for: Characterizing Genetic, Epigenetic, Nutritional, and Clinico-Biochemical Profile of Women With Polycystic Ovary Syndrome: A Case–Control Study
Source: J Nutr Metab. 2025 May 30;2025:8817919. doi: 10.1155/jnme/8817919 (PMC12143951; doi:10.1155/jnme/8817919)
Supplement: Supporting Information — Additional supporting information can be found online in the Supporting Information section. [file 8817919.f1.docx]

**Supplementary Table 1:** Subgroup differences between cases and controls.

| **Variable** | | **Cases (n=66)**  **n (%)** | **Controls(n=69) n (%)** | **P-value**  **(Difference between cases and controls)** |
| --- | --- | --- | --- | --- |
| **Glucoregulatory markers** | | | | |
| **Glucose** | **Less than normal** (< 70 mg/dl) | 21 (31.8%) | 19 (27.5%) | 0.383 |
|  | **Normal** (70-110 mg/dl) | 40 (60.6%) | 47 (68.1%) |  |
|  | **More than normal** (> 110 mg/dl) | 5 (7.6%) | 2 (2.9%) |  |
| **Insulin** | **Normal** (< 10 μIU/ml) | 33 (50.0%) | 42 (60.9%) | 0.305 |
|  | **Mild resistance** (10-14 μIU/ml) | 11 (16.7%) | 11 (15.9%) |  |
|  | **Moderate to severe** (> 14 μIU/ml)  **resistance** | 22 (33.3%) | 15 (21.7%) |  |
| **HOMA-IR** | **Normal** (< 1.9) | 34 (51.5%) | 44 (63.8%) | 0.085 |
|  | **Not normal** (> 1.9) | 32 (48.5%) | 24 (34.8%) |  |
| **Lipid profile** | | | | |
| **LDL** | **Optimal** (< 100 mg/dl) | 21 (31.8%) | 22 (31.9%) | 0.941 |
|  | **Border line** (100-160 mg/dl) | 38 (57.6%) | 40 (58.0%) |  |
|  | **High** (> 160 mg/dl) | 7 (10.6%) | 6 (10.1%) |  |
| **HDL** | **Low** (< 35 mg/dl) | 3 (4.5%) | - | 0.059 |
|  | **Normal range** (35-79.5 mg/dl) | 62 (93.9%) | 63 (91.3%) |  |
|  | **High** (> 79.5 mg/dl) | 1 (1.5%) | 5 (7.2%) |  |
| **TG** | **Desirable** (< 150 mg/dl) | 48 (72.7%) | 58 (84.1%) | 0.057 |
|  | **Borderline** (150-160 mg/dl) | - | - |  |
|  | **High** (> 160 mg/dl) | 18 (27.3%) | 10 (14.5%) |  |
| **TC** | **Desirable** (< 200 mg/dl) | 33 (50.0%) | 39 (56.5%) | 0.566 |
|  | **Borderline** (200-240 mg/dl) | 19 (28.8%) | 19 (27.5%) |  |
|  | **High** (> 240 mg/dl) | 14 (21.2%) | 10 (14.5%) |  |
| **Vitamin D and hormonal markers** | | | | |
| **Vitamin D status** | **Deficient** (< 50 nmol/L) | 39 (59.1%) | 39 (56.5%) | 0.242 |
|  | **Insufficient** (50 - <75 nmol/L) | 16 (24.2%) | 11 (15.9%) |  |
|  | **Sufficient (Adequate)** (75 - 250 nmol/L) | 10 (15.2%) | 19 (27.5%) |  |
|  | **Toxicity** (> 250 nmol/L) | 1 (1.5%) | - |  |
| **TSH** | **Less than normal** (< 0.34 μIU/ml) | - | 1 (1.4%) | 0.613 |
|  | **Normal** (0.34 – 5.6 μIU/ml) | 64 (97.0%) | 65 (94.2%) |  |
|  | **More than normal** (> 5.6 μIU/ml) | 2 (3.0%) | 2 (2.9%) |  |
| **LH** | **Low** (Less than 10 mIU/ml) | 47 (71.2%) | 52 (76.5%) | 0.310 |
|  | **High** (More than or equal to 10 mIU/ml) | 19 (28.8%) | 16 (23.5%) |  |
| **LH / FSH ratio** | **Low** (Less than 1) | 33 (50.0%) | 27 (39.7%) | 0.150 |
|  | **Normal** (1.0 – 1.5) | 11 (16.7%) | 21 (30.9%) |  |
|  | **High** (More than 1.5) | 22 (33.3%) | 20 (29.4%) |  |

*CRP: C-reactive protein, HOMA-IR index: Homeostatic model assessment of insulin resistance, QUICKI: Quantitative Insulin Sensitivity Check Index. LDL: Low-density lipoprotein, HDL: High-density lipoprotein, TG: Triglyceride, TC: Total cholesterol, VLDL: Very low-density lipoprotein.

*Vitamin D: (25-OH-Cholecalciferol), FSH: Follicle stimulating hormone, TSH: Thyroid stimulating hormone, LH: Luteinizing hormone.
